# Determination of *Leptospira borgpetersenii* serovar Javanica and *Leptospira interrogans* serovar Bataviae as the persistent *Leptospira* serovars circulating in the urban rat populations in Peninsular Malaysia

**DOI:** 10.1186/s13071-016-1400-1

**Published:** 2016-03-01

**Authors:** Douadi Benacer, Siti Nursheena Mohd Zain, Shin Zhu Sim, Mohd Khairul Nizam Mohd Khalid, Renee L. Galloway, Marc Souris, Kwai Lin Thong

**Affiliations:** Institute of Biological Sciences, Faculty of Science, University of Malaya, Kuala Lumpur, Malaysia; Institute of Mathematical Sciences, Faculty of Science, University of Malaya, Kuala Lumpur, Malaysia; Lee Kong Chian Faculty of Engineering and Science, Universiti Tunku Abdul Rahman, Kuala Lumpur, Malaysia; Bacteriology Unit, Institute for Medical Research, Ministry of Health, Kuala Lumpur, Malaysia; Bacterial Special Pathogens Branch, Centers for Disease Control and Prevention, Atlanta, Georgia; UMR_D 190 “Emergence des Pathologies Virales”, IRD Aix- Marseille University EHESP, Marseille, France

**Keywords:** *Leptospira*, Leptospirosis, Rodents, Outbreaks

## Abstract

**Background:**

Leptospirosis is an emerging infectious disease of global significance, and is endemic in tropical countries, including Malaysia. Over the last decade, a dramatic increase of human cases was reported; however, information on the primary vector, the rat, and the *Leptospira* serovars circulating among the rat population is limited. Therefore, the present study was undertaken to isolate *Leptospira* and characterise the serovars circulating in the urban rat populations from selected main cities in Peninsular Malaysia.

**Methods:**

Rat trappings were carried out between October 2011 to February 2014 in five urban cities which were chosen as study sites to represent different geographical locations in Peninsular Malaysia. Microscopic agglutination test (MAT) and PCR were carried out to identify the Leptospiral serogroup and determine the pathogenic status of the isolates, respectively while pulsed-field gel electrophoresis (PFGE) and random amplified polymorphic DNA (RAPD)-PCR were used to characterize the isolates.

**Results:**

Three rat species were identified from the three hundred and fifty seven rats captured with *Rattus rattus*, being the dominant rat species (285, 80 %) followed by *Rattus norgevicus* (53, 15 %) and *Rattus exulans* (19, 5 %). Only 39 samples (11.0 %) were positive by culture and further confirmed as pathogenic *Leptospira* by PCR. Significant associations were shown between host infection with locality, season, host-age and species. Based on MAT, two serogroups were identified in the population namely; *L. borgpetersenii* serogroup Javanica (*n =* 16) and *L. interrogans* serogroup Bataviae (*n =* 23). Pulsed-field gel electrophoresis (PFGE) distinguished the two serovars in the urban rat populations: *L. borgpetersenii* serovar Javanica (41 %), and *L. interrogans* serovar Bataviae (59 %). RAPD-PCR yielded 14 distinct patterns and was found to be more discriminative than PFGE.

**Conclusions:**

This study confirms two *Leptospira* serovars circulating among the urban rats population in Peninsular Malaysia namely; *L. borgpetersenii* serovar Javanica and *L. interrogans* serovars Bataviae. Despite the low number of isolates obtained from the rat population, this study suggests that rodent control programs and disease surveillance may help to reduce the possible risk of disease transmission.

## Background

Leptospirosis is one of the most widespread zoonotic bacterial diseases with a worldwide distribution [1]. It is caused by the pathogenic species from the genus of *Leptospira*. and affects both humans as well as domestic and wild animals. It is maintained and spread throughout the environment through the urine of infected animals. Human infection occurs through exposure to water or soil contaminated with urine of infected animals or through direct contact with contaminated urine [2]. Leptospirosis occurs mainly in tropical and sub-tropical regions where environmental and socioeconomic conditions for its transmission and survival are particularly favourable [3, 4]. Leptospires thrive in warm, moist soil and immersion in water for long periods [5]. At present, leptospirosis is commonly associated with recreational activities in wild environments [6]. Certain occupations and recreational activities are at higher risk of contracting the infection due to the nature of these activities and work environment. In Malaysia, several outbreaks were related to recreational activities [7, 8]. Leptospirosis is also known as an occupational disease, commonly occurring among farmers, veterinarians, fishermen, livestock and abattoir workers [1, 9].

Natural reservoirs of leptospires are rodents and a large variety of other feral and domestic animals. Many serotypes occur predominantly in select mammalian hosts; however, the distribution of a specific serotype in a select host is not exclusive.

In Malaysia, the first study on a leptospirosis was recovered from black rats [10] and the rodents are now considered the principal maintenance hosts of *Leptospira* and the carriers for pathogenic *Leptospira* serovars. To date more than 37 *Leptospira* serovars from 13 different serogroups have been identified in Malaysia with more than half found to be carried by rodents [11].

Locally, dramatic increases in human cases were reported over the last decade. In 2013 there were 4,457 cases with 71 deaths marking an increase in disease incidence from 12.5 per 100,000 population in 2012 to 15.0 per 100,000 population the following year (Data extracted from official report from the Ministry of Health Malaysia). Recently an outbreak occurred in Kelantan State where more than 94 human leptospirosis cases were reported after the floods that hit the eastern side of the Peninsular between 1 and 18 of January 2015 [12].

The majority of cases in this country were related to the exposure of humans to environment contaminated by *Leptospira* spp. [13, 14]. However, there is little information regarding the primary carrier vector, the rat and the risk to human transmission in urban settings of Malaysia.

A recent study revealed two predominant pathogenic *Leptospira* serovars from two species; *Leptospira borgpetersenii* serovar Javanica and *Leptospira interrogans* serovars Bataviae circulating in the two dominant rat species, *Rattus rattus* and *Rattus norvegicus* in Kuala Lumpur [11]. The presence of pathogenic *Leptospira* thriving in an urban rat population, facilitated with abundance of food and improper garbage management, in addition to rapid urbanisation and growing of slums with inadequate infrastructures (sewage, water) could ultimately bring rodents in close proximity to humans. Therefore the objective of this study was to determine *Leptospira* serovars circulating in several urban rat populations in Peninsular Malaysia.

Five different States representing North (Penang, Perak), East (Pahang), West (Selangor) and South (Malacca) parts of Peninsular Malaysia were selected as study sites based on the disease incidence over the past few years [15]. The identification of *Leptospira* and determination of its pathogenic status of the isolates were carried using MAT and PCR while PFGE and RAPD-PCR were used to analyze the genetic relatedness of the recovered isolates.

## Methods

### Ethics statement

This study was approved with the ethics reference no. ISB/31/01/2013/SNMZ (R) by the Institutional Animal Care and Use Committee, University of Malaya, Malaysia (UM IACUC). This study did not involve any endangered or protected species.

### Choice of the study sites

**Fig. 1:**
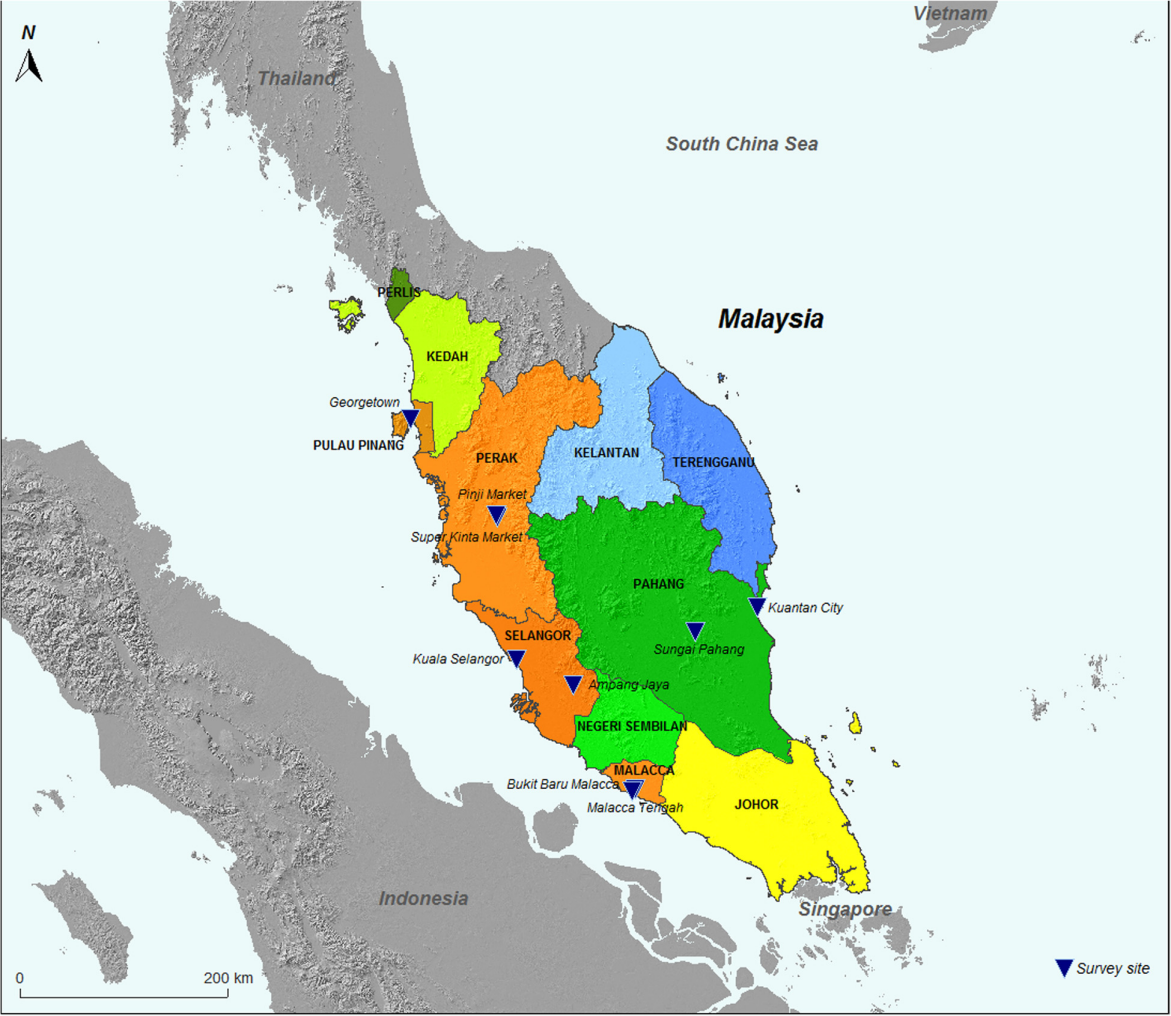
The map showing the five States selected for trapping in Peninsular Malaysia

Several locations were selected as study sites to represent different geographical locations in Peninsular Malaysia namely; Penang and Perak (North), Selangor (East), Malacca (South) and Pahang (West) (Fig. 1). The trappings were conducted with the assistance of the municipality from each city as part of the vector control programme. The sites chosen were based on the suitability of the habitat for rats to forage, breed and the risk of transmission. This included wet markets, food courts, restaurants and hawker stalls with abundance of leftovers (food remnants). In addition, high density residential areas particularly from the lower income bracket and nearby open wet markets providing fresh produce to the community and poor garbage management, were selected as these sites attracted rodents and stray animals to breed and to source for food. All sites were characterized by tropical climate and high humidity throughout the year with temperatures ranging between 30 °C and 36 °C with heavy rainfall coinciding with the monsoon season. For this purpose, season is divided into wet and dry seasons for each year with dry months falling between March – September and wet season between October – February.

### Trapping and host identification

Trapping was conducted over a period of 6 days and 5 nights for each session with a total of 36 trapping sessions conducted between October 2011 and February 2014. The wire traps (18 x 12 x 28 cm) were placed in each site with baits such as dry fish, breads, and peanut butter. The traps were placed in the evening and collected early morning before the market was opened to the public. The captured rats were placed in black plastic bags to reduce the stress and transported to the laboratory for examination. The rats were then euthanized and morphometric measurements recorded [11]. Age and species identification were carried out according to Medway [16] based on the phenotypic characteristics such as fur color (ventral and dorsal), body weight, hind foot and head-body length.

### Isolation of *Leptospira* and dark field microscopy examination

*Post mortem* examination was performed to retrieve selected organs such as blood, livers and kidneys. The urine was collected *via* direct puncture of the bladder and then cultured into the modified semi-solid EMJH medium. Kidney tissue samples were removed using a sterile blade, and a small piece of tissue was placed in a sterile syringe without a needle and squeezed into the EMJH medium supplemented with 5-fluorouracil (Merck, Germany). All the inoculated media were incubated aerobically at 30 °C, examined under a dark field microscope for the presence of *Leptospira* at 10-day intervals for a period of 3 months. Samples that failed to show any evidence of growth after 3 months were considered negative.

### PCR detection and confirmation of leptospiral isolates by PCR

DNA was extracted from 7 day-old culture media using Wizard™ Genomic DNA Purification Kit (Promega, USA) following the manufacturer’s instructions. The quantity of DNA was measured by Biophotometer (Eppendorf, Germany).

PCR primers, LG1/LG2 [(5^′^-CGG TGA AAT GCG TAG ATA TC-3^′^) and (5^′^- CGG TTT GTC ACC GGCAGT TC-3^′^)] were designed in-house to target the 16S rDNA gene to confirm the genus of *Leptospira* isolates. The cycling conditions consisted of an initial denaturation at 94 °C for 3 min, 35 cycles each of 94 °C for 1 min, 57 °C for 1 min, 72 °C for 2 min, and further extension at 72 °C for 10 min. To determine the pathogenic status of the isolates, the published G1/G2 primers were used [(5^′^-CTG AAT CGC TGT ATA AAA GT-3^′^) and (5^′^-GGA AAA CAA ATG GTC GGA AG-3^′^)], which target the *secY* gene among the pathogenic species except for *L. kirschneri* [17]. The cycling conditions consisted of an initial denaturation at 94 °C for 10 min followed by 35 cycles of 94 °C for 1 min, 55 °C for 1 min, 72 °C for 1 min, and final extension at 72 °C for 5 min. In both PCRs, the reactions were done in a final volume of 25 μl containing 1X PCR buffer, 1.5 mM MgCl_2_, 200 μM each of dNTPs, 0.3 μM of each primer, 1 U of *Taq* DNA polymerase (Intron, Biotechnology, South Korea) and 100 ng of DNA template. The PCR products were analyzed by electrophoresis through a 1 % agarose gel (Promega, Madison, USA).

*DNA sequencing.* PCR products from representative isolates were verified by DNA sequencing. The amplicons were purified using DNA purification kit (Qiagen, Germany) and sent to a commercial facility for sequencing (First BASE, Pte. Ltd., Singapore). The resulting DNA sequence data were compared with the GenBank database using the BLAST algorithm available at web site (http://www.ncbi.nlm.nih.gov).

### Microscopic agglutination test (MAT)

Live isolates were used for MAT as described by World Health Organization [18]. The isolates were cultured into liquid medium supplemented with 1.0 % rabbit serum to increase bacterial density [11]. The antisera used in this study were raised against serovars Sejroe (M84), Javanica (Veldrat Batavia 46), Canicola (Hond Ultrecht IV), Hebdomadis (Hebdomadis), Pomona (Pomona), Hardjo (Hardjoprajitno), Australis (Ballico), Bataviae (Swart), Pyrogenes (Salinem), Icterohaemorrhagiae (RGA), Autumnalis (Akiyami A), Grippotyphosa (Mandemakers).

### Pulsed field gel electrophoresis

PFGE was performed according to protocol [19] with minor modifications. Leptospiral DNA embedded into agarose plugs were digested with 10 U of restriction enzyme *Not*I (Promega, Madison, USA) at 37 °C. The restriction fragments obtained were separated by electrophoresis in 0.5X TBE buffer, for 24 h at 14 °C in a CHEF Mapper system (Bio-Rad, USA) using pulsed times of 2.2 to 35 s. *Xba*I-digested *Salmonella* Braenderup H9812 was used as the DNA size marker. PFGE data were analyzed using BioNumerics Version 6.0 (Applied Maths, Belgium) software. Clustering was based on the unweighted pair group average method (UPGMA) with the position tolerance of 1.0.

### Subtyping of *Leptospira* Isolates by using RAPD-PCR

RAPD-PCR fingerprinting was performed according to Ramadass et al. [20] using B11 (5′-CCG GAA GAA GGG GCG CCA T-3′) and B12 (5′-CGA TTT AGA AGG ACT TGC ACA C-3′) primers with some modifications. The reaction was carried out in a final volume of 25 μl containing 1X PCR buffer, 1.5 mM MgCl_2_, 200 μM each of dNTPs, 0.3 μM of each primer, 1 U of *Taq* DNA polymerase (Intron, Biotechnology, South Korea) and 100 ng of DNA template. The cycling conditions consisted of two cycles of denaturation at 95 °C for 5 min, annealing of primers for 5 min at 40 °C, and extension for 5 min at 72 °C. The subsequent 35 cycles consisted of denaturation for 1 min at 95 °C, annealing of primers for 1 min at 60 °C, and extension for 3 min at 72 °C, with a final extension step for 10 min during the last cycle. PCR products were analyzed by electrophoresis through a 1 % agarose gel (Promega, Madison, USA).

#### Statistical analysis

All statistical analyses were performed using the logistic regression model in R software (version 3.1.1) [21]. The infection status of individual rats was investigated based on the effects of host-age, host-sex, host-species, season and location. In our study, the samples that showed the typical morphology and the characteristic motility of *Leptospira* genus under the dark field microscope from urine and kidney samples and confirmed by MAT and PCR were considered as positive samples. Further sequencing of the PCR products were done to confirm the results. Akaike’s Information Criterion (AIC) was used for the selection of the best model. It is defined as *AIC = 2 k*–*2InL* where *k* is the number of parameters in the model and *InL* is the maximum of the log-likelihood function for the data [22]. The model with the lowest AIC value was selected. The significance tests for the individual explanatory variables were performed by computing Wald statistics. A small *p*-value (<0.05) of the Wald test indicates a detection of a statistical significance variable in our model.

## Results

A total of 357 rodents captured were composed predominantly of *Rattus rattus* 285 (80 %), followed by *Rattus norvegicus* 53 (15 %) and *Rattus exulans* 19 (5 %) (Table 1). The highest capture was from the vicinity of Pasar Super Kinta in Ipoh, Perak (105 rats, 29 %), followed by Taman Bukit Baru residential area in Malacca with 95 (27 %) rats. Based on the host-age and sex, more adults 265 (74 %) were captured than juveniles 92 (26 %) with more females (216, 61 %) compared to males (141, 39 %). Isolation of *Leptospira* was achieved from 39 captured rats (11.0 %) which were recovered from 35 urine and 4 kidney samples. The cultures showed the typical morphology and the characteristic motility of species of *Leptospira* by dark field microscopy.

**Table 1:** Summary of the rodent population according to rat species, host-sex and host-age from five states in peninsular Malaysia

States	Location	Host-species			Host-sex		Host-age		Season		Source		Result	
		RR	RN	RE	Male	Female	Adult	Juvenile	Dry	Wet	Urine	Kidney	Negative	Positive
Selangor	Kuala Selangor	10	2	0	4	8	6	6	7	5	8	4	12	0
	Ampang Jaya	22	24	4	17	33	38	12	0	50	14	36	47	3
Penang	Georgetown	4	11	0	10	5	15	0	0	15	8	7	10	5
Pahang	Kuantan city	31	10	12	24	29	48	5	18	35	25	28	41	12
	The Sungai Pahang	18	0	3	9	12	15	6	21	0	5	16	21	0
Malacca	Taman Bukit Baru	90	5	0	31	64	73	22	95	0	82	13	90	5
	Melaka Tengah district	2	1	0	1	2	3	0	3	0	3	0	2	1
Perak	Super Kinta market (Ipoh)	105	0	0	43	62	64	41	14	91	87	18	92	13
	Pinji market (Ipoh)	3	0	0	2	1	3	0	0	3	3	0	3	0
Total (Number of infected rats)		285 (24)	53 (8)	19 (7)	141 (19)	216 (20)	265 (38)	92 (1)	158 (7)	199 (32)	235	122	318	39

*RR* *Rattus rattus*

*RN* *Rattus norvegicus*

*RE* *Rattus exulans*

The statistical analysis revealed that the infection status of individual rats depends on the host-age, host-species, season and location. Note that the variable host-sex was excluded because the Wald test for significance of this variable yielded *p*-value of 0.93. This indicates that the variable host-sex appears to be redundant in the model.

From Table 2, the generalized linear model analysis shows that the odds of positive infection were 93 % less likely among juvenile rats as compared to adult rats (odds ratio = 0.07) after adjusting for host-species, season and location. For host-species, the rat species *R. exulans* had a 5.58 times greater odds of positive infection than the rat species *R. rattus* after adjusting for host-age, season and location. Meanwhile, the variable season was found to be statistically significant (*p*-value = 0.04). The odds of infected rats were about 10 times higher among rats captured during the wet season as compared to rats captured during the dry season (odds ratio = 9.97) after adjusting for host-age, host-species and location. This indicates that rats captured during the wet season were more likely to be infected than the rats captured during the dry season. Also, the odds of infected rats found in locations Penang, Pahang, Malacca and Perak were higher than the location Selangor after adjusting for host-age, host-species and season.

**Table 2:** Generalized linear model of urban rats infections from Peninsular Malaysia (October 2011 to February 2014) by *Leptospira* with Binomial distribution and logit link function

Variables	Estimate	(Standard error)	*p*-values
(Intercept)	−5.18	1.23	< 0.001
Host-age: juvenile	−2.66	1.04	0.01
Host-species: *Rattus norvegicus*	0.28	0.62	0.65
Host-species: *Rattus exulans*	1.72	0.76	0.02
Location: Penang	1.97	0.83	0.02
Location: Pahang	1.47	0.75	0.05
Location: Malacca	2.68	1.29	0.04
Location: Perak	1.53	0.77	0.05
Season: wet	2.30	1.09	0.04

Null deviance: 246.28 on 356 degrees of freedom

Residual deviance: 197.83 on 348 degrees of freedom

AIC: 215.83

Genus confirmation by PCR using the designed primers LG1/LG2 showed that all 39 isolates gave the expected band sized 483 bp, indicating that all the positive isolates were *Leptospira* spp. (99 % similarity, accession no. KC662455, JQ988849). In addition, all isolates were pathogenic species as shown by the band sized 285 bp amplified by the primers G1/G2 which targets *secY* genes (99 % similarity, accession numbers: EU358040, DQ882852).

The identification of the serogroup of the isolates was carried out using the 13 different hyperimmune antisera. Results showed 23/39 isolates reacted by agglutination against the serovar Bataviae antisera (titer > 1:400), while only 16 isolates reacted towards the serovar Javanica (titer > 1:400). All 13 isolates from Ipoh, Perak were identified from the serogroup of Javanica, while 6 isolates from Malacca belong to Bataviae serogroup. The majority of isolates from Kuantan, Pahang State (11 isolates) belong to Bataviae serogroup while only one was from Javanica serogroup. Two isolates from Ampang Jaya in Selangor were from Bataviae serogroup, while only one from Javanica serogroup. Four isolates from Penang were from Bataviae serogroup and only one from Javanica serogroup.

**Fig. 2:**
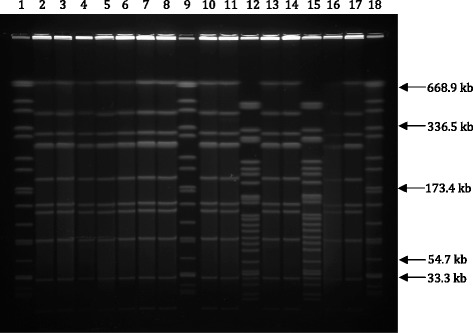
Representative PFGE patterns of *Not*I digested chromosomal DNA of *Leptospira* isolates from urban rats of Peninsular Malaysia. Lanes 1, 9, 18: *XbaI* digested chromosomal DNA of *Salmonella* H9812 marker strain; Lanes: 2–8: *L*. Bataviae RK84; *L*. Bataviae RK87; *L*. Bataviae RK94; *L*. Bataviae RK100; *L*. Bataviae RK102; *L*. Bataviae RK108; *L*. Bataviae RK109; Lanes: 10–11: *L*. Bataviae RP6; *L*. Bataviae RP11; Lane: 12: *L*. Javanica RI26; Lanes: 13–14: *L*. Bataviae RM15; *L*. Bataviae RM29; Lane: 15: *L*. Javanica RI42; Lanes: 16–17: *L*. Bataviae RA50; *L*. Bataviae RM39

PFGE of *Not*I-digested chromosomal DNA subtyped the 39 isolates into 2 distinct PFGE profiles LNot01, and LNot02. The number of DNA fragments generated ranged from 10 to 23 with sizes ranging approximately from 28 kb to 706 kb (Fig. 2). Limited genetic diversity was found among the isolates.

The 2 PFGE profiles generated were compared to the *Not*I patterns from the database provided by the Centers for Disease Control and Prevention (CDC), USA. The results showed that LR01 were similar to *L. interrogans* serovar Bataviae, whereas LR02 were similar to *L. borgpetersenii* serovar Javanica*.*

RAPD-PCR using the primers B11 and B12 subtyped the 39 positive isolates into 14 different patterns. All the strains were type-able by using RAPD-PCR and the band patterns were highly variable (polymorphism). Clear genomic patterns were obtained for all strains, even though some variation of band intensity could be observed.

**Fig. 3:**
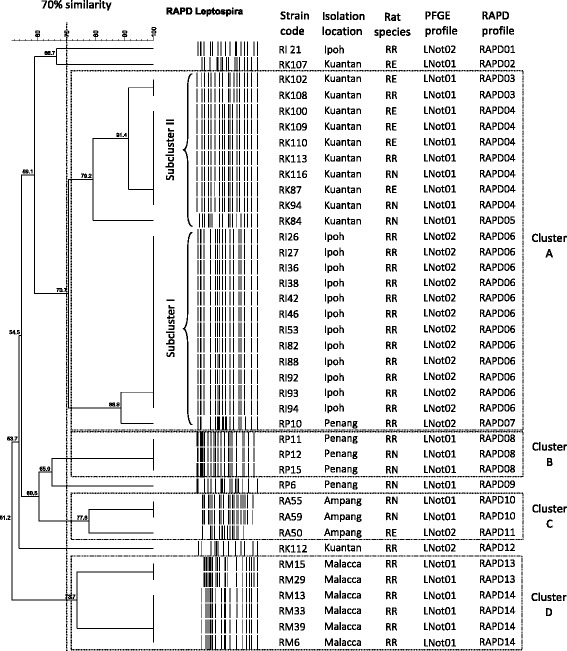
RAPD dendrogram cluster analysis generated using Bionumeric Version 6.0 (Applied Maths, Belgium) software and unweighted pair group arithmetic means methods (UPGMA) of *Leptospira* strains

Each strain contained between 15 to 27 bands ranging in size from 200 bp to 2000 bp. Bands below 200 bp and above 2100 bp were not included in the analysis. High genetic diversity was observed among the isolates from Kuantan, Pahang, as the 13 isolates gave six different RAPD patterns. A limited genetic diversity was observed among the isolates isolated from Malacca and Ipoh, Perak. The three isolates from Ampang Jaya in Selangor and the five isolates from Penang gave three different patterns, respectively. The dendrogram generated four major clusters, namely cluster A, B, C and D at 70 % of similarity (Fig. 3). Cluster A consisted of 23 isolates and it is further divided into two subclusters (subcluster I and II). Subcluster I consisted of 13 isolates recovered from *R. rattus* and had the same PFGE profile (LNot02) in both Ipoh (11 isolates) and Penang (2 isolates). Subcluster II consisted of ten isolates recovered from *R. exulans* (5), *R. norvegicus* (3) and *R. rattus* (2). All the ten isolates had the same PFGE profile (LNot01).

Cluster B consisted of three isolates from Penang which had the same PFGE profile (LNot01) and isolated from two *R. norvegicus* and one *R. rattus*. Cluster C consisted also of three isolates from Ampang, Selangor and had two different PFGE profiles (LNot01, LNot02). Two of them were isolated from *R. novegicus* and one was from *R. exulans*. Cluster D consisted of six isolates which were isolated from *R. rattus* captured in Malacca and had the same PFGE profile (LNot01) (Fig. 3).

## Discussion

Rapid urbanization and urban poverty have led to the dramatic growth of slum settlements in many low and middle-income countries [23]. These locations are often characterized by poor infrastructure with an ineffective irrigation system, garbage management system and sanitation facilities that promote proliferation of rodents and poses the risk of rodent-borne transmission. Rats are known as the source of a number of pathogens responsible for significant human morbidity and mortality in many cities around the world [24]. They act as the primary hosts of *Leptospira* spp. and are well recognized as the most significant mammal species in maintaining and disseminating of leptospires worldwide [25].

In Malaysia, leptospirosis is an endemic disease and recently there has been an increase in the number of reported human cases. Hence it is important to investigate the role of the rats in the dissemination of disease in urban settings. Information with regards to the disease incidence and leptospiral serovars circulating among the urban rat population in the Peninsular Malaysia is essential to have a better understanding of how these reservoirs contribute to disease transmission to humans.

Five major states representing different unique geographical locations in Peninsular Malaysia were selected for rat trapping based on the high incidence of human leptospirosis cases between 2004 and 2012 “unpublished data”. Sites were also chosen based on presence of recent rat activity in public spaces. Wet markets were an ideal site for both rats and stray animals to forage since food leftovers are aplenty and consequently such places become their breeding grounds. On the other hand, the presence of rats was also observed in slum residential areas due to improper garbage disposal and poor irrigation system with open sewers. These conditions have created favorable habitat for the survival of the host reservoir and subsequently the contamination of environmental waters and soils *via* their excreta and urine.

In this study, *R. rattus* was the dominant rat species captured in the five States (*p* < 0.001) followed by *R. norvegicus*. This result concurred with our previous study [11] indicating that both species are the two dominant rat species in the urban areas of Peninsular Malaysia. Both species are commensal rats and are generally found living closely to human habitation and dependent on human wastes for food, water and space for shelter [26, 27]. Although the Norway rat (*R. norvegicus*) is capable of living in isolation, the convenient human environment is preferable to them and as a result they were found less commonly in the rural environment. A small population of *Rattus exulans* were captured close to the forest fringes of the urban cities. *R. exulans* or known as Polynesian rat is the third most widespread species rat in the world after *R. rattus* and *R. norvegicus.* This species generally inhabits rural scrub, rubber and coconut palm plantations. However, the presence of this species in the cities could be explained by the migrating rats for scavenging as well as the rapid urbanization and the deforestation at the fringes of the cities which have put humans in contact with more animal reservoirs.

The highest number of captured rats was in Ipoh, Perak State. The two trapping locations (Pinji and Super Kinta) were wet markets that sold fresh produce with plentiful resources for the rodents to forage. Both markets lacked proper maintenance with water in containers left unprotected overnight and open sewers and drains that were full of rubbish. These conditions not only attracted rats to forage at night but also pose the risk of possible contamination with their urine, serving as a source of infection for both man and animals*.*

Taman Bukit Baru in Malacca State recorded the second highest rat capture and this site comprised of residential area with old buildings with poor irrigation and improper rubbish management system. Rats were frequently observed roaming near rubbish dumps close to the housing area. This also poses a high risk of exposure to humans especially children, who play in the surrounding vicinity as living in close proximity to accumulated garbage had been found to significantly increase the risk to leptospirosis [28].

The levels of infection found in the rodent population in Peninsular Malaysia were low (11 %). However these results were comparatively higher to our previous study (6.7 %) in the capital city of Kuala Lumpur [11]. Despite the low infection rate reported, the high pathogenicity of these serovars raises concern of public health risks caused by the transmission of leptospirosis. Ivanova et al. [29] noted similar infection rates in rodent and shrews from several locations in Cambodia while Cosson et al. [30] noted low *Leptospira* prevalence among trapped rodents from seven localities in Southeast Asia (Thailand, Laos and Cambodia).

For the three species captured*,* infections were more prevalent in *R. exulans* (38 %) compared to *R. norvegicus* (18 %) and *R. rattus* (7 %). To date, *Leptospira* spp. has been successfully isolated from more than ten rat species including *R. exulans* [31–33] in Malaysia. The finding is also in agreement with studies conducted by Wangroongsarb et al. [34] on 1,164 rodents captured from ten epidemic states in Thailand and where they noted highest infection rate in *R. exulans*. However, their results did not concur with other studies [29, 35].

Host age played an important role in influencing the infection with more adult rats being significantly infected with *Leptospira* than immature rats and this is in agreement with other studies [11, 25, 36]. Physiology, immunology and behavioral characteristics related to age are reported to play an important role in the transmission of the infections [35]. Adult rats forage further and have a more dynamic movement which put them at higher risk of exposure to the infection compared to the juvenile or newborns that are generally confined to the nesting burrows [37]. Aggressive behaviour of the adult rats such as fighting and biting is known to facilitate the transmission of leptospiral infection [32] as reported by Himsworth et al. [38] that the weight, body fat and bite wounds common among the adult rats increases the probability of leptospiral infection.

A similar carriage between males and females rats was recorded in this study with no correlation between infection rates with host-sex. This finding differed from other studies where significantly more males were infected [11, 33]. In contrast, Agudelo-Florez et al. [39] reported no relationship between host-sex, host-age or host health with the disease prevalence.

This investigation also recorded the influence of season on the disease prevalence with significantly more rats infected during the wet season as observed in the States of Pahang, Penang and Perak and this result concurred with other studies [25, 29]. Heavy rainfall during the monsoon months readily facilitates the transmission of waterborne bacteria including *Leptospira* spp. as the moisture and humidity increase and the availability of fresher and cleaner water bodies to thrive in. The combination of all these factors can lead to a more favorable environment for the survival of leptospires [25]. In contrast, initial *Leptospira* survival is likely to be poor in the dry season as rodent urine quickly dries up on dry ground. The numbers of leptospirosis-infected rodents and the abundance of leptospires in the environment are both potential indicators of risk of leptospirosis infection to humans [40].

Several studies reported the low sensitivity of the culture technique in the detection of *Leptospira* spp. compared to other techniques such as PCR, immunofluorescence and nucleic acid hybridization [41, 42]. Besides this, the success of the isolation step is influenced by various factors such as the number of organisms per inoculation, the type of media used, and also the type of specimen. The difficulty in the isolation of *Leptospira* and the slow growth of this fastidious organism made the culture technique time consuming with low sensitivity.

Presently, PCR analysis is increasingly used as a promising tool in the diagnosis of leptospirosis due to the high rapidity, sensitivity and specificity in the detection of *Leptospira* in different specimens including clinical, animal and environmental samples [14, 43, 44].

In the present study, PCR was able to successfully determine the genus and pathogenic status of the isolates. To determine the *Leptospira* genus of the isolates, the designed primers LG1/LG2 yielded a product band size of 483 bp from cultured positive samples. These primers were designed from *rrs* genes and expected to amplify leptospiral DNA from both saprophytic and pathogenic species. The specificity and the sensitivity of these primers were checked using 12 different *Leptospira* reference strains and 10 non-leptospiral bacteria. All the 39 isolates were confirmed as *Leptospira* genus. The pathogenicity status of the isolates was checked using G1/G2 primers. The result showed that all the confirmed *Leptospira* isolates were pathogenic.

The serogroups of the 39 isolates were determined using 13 different antisera and based on MAT technique. Only 2 serogroups were identified; with serogroup Bataviae (59 %) marginally higher than serogroup Javanica (41 %) with both serogroups previously reported in Kuala Lumpur city [11]. The persistence of these two serogroups circulating in the rat population indicates its role as a maintenance host in the transmission of *Leptospira*. Shafei et al. [45] reported two serogroups, Bataviae and Javanica predominant among town service workers in the north-eastern state of Malaysia.

PFGE of *Not*I-digested *Leptospira* DNA gave two profiles and the results were in concordance with MAT results. With reference to PFGE profiles available in the leptospiral database at CDC, we were able to identify *L. interrogans* serovar Bataviae in 23 isolates and *L. borgpetersenii* serovar Javanica in 16 isolates. Hence, PFGE could be used to complement serogrouping. However, PFGE was not discriminative enough to distinguish isolates from different sources. Therefore, an apparent lack of genetic diversity was observed within the members of serovars of Javanica and Bataviae. All 23 Bataviae isolates had the same PFGE profiles, despite being collected from different locations in four different States including Malacca, Pahang, Penang and Selangor, different rat species and at different sampling times. Similarly, all the 16 Javanica, which originated from different geographical locations were indistinguishable. This was in agreement with the observation of the previous studies [11, 19] that PFGE was unable to discriminate strains within the same serovars such as Icterohaemorragiae and Copenhageni of *L. interrogans* species. However, PFGE showed a high discriminatory ability to differentiate different serovars of *L. borgpetersenii* and *L. interrogans.*

Hence, a second subtyping method, RAPD-PCR was used to improve the discrimination. In this study a greater genetic variation was observed among the members of the same serovar as the 39 isolates were represented by 14 different patterns. Some of isolates represented the same serovar isolated from different locations gave different RAPD patterns. Our study was in agreement with several previous studies [46, 47]. RAPD-PCR is shown to be a useful technique in epidemiological investigation of leptospirosis. In several studies, RAPD-PCR had the ability to discriminate between the strains at species and even at serovars level [20, 48]. Hence, based on discriminatory power, RAPD is more discriminative compared to PFGE which failed to distinguish strains within the same serovar. However, the reproducibility of the PCR fingerprinting is moderate, which makes pattern analysis more difficult and tedious as compared to PFGE. PFGE examines the genetic variation throughout the genome and is highly reproducible, which makes it the “gold standard” molecular typing of *Leptospira* spp. [19]. RAPD fingerprint could be an alternative tool in subtyping of *Leptospira* isolates, as it is easier and could generate results more rapidly than PFGE.

## Conclusions

*L. borgpetersenii* serovar Javanica and *L. interrogans* serovar Bataviae were predominant among the urban rats in all the States studied which concurred with our previous study of the urban rats in Kuala Lumpur [11]. Despite the low disease prevalence, this finding highlights the risk of close proximity of this maintenance host to humans in urban settings. Therefore, assessment and regular monitoring of infections by wild rodents is necessary to address an important health problem and provide conclusions that can inform the design of effective public health policy.

The findings and conclusions in this report are those of the authors and do not necessarily represent the official position of the Centers for Disease Control and Prevention.
